# Artificial and Natural Sialic Acid Precursors Influence the Angiogenic Capacity of Human Umbilical Vein Endothelial Cells

**DOI:** 10.3390/molecules18032571

**Published:** 2013-02-26

**Authors:** Nils B. Bayer, Uwe Schubert, Zehra Sentürk, Silvia Rudloff, Sandra Frank, Heike Hausmann, Hildegard Geyer, Rudolf Geyer, Klaus T. Preissner, Sebastian P. Galuska

**Affiliations:** 1 Institute of Biochemistry, Faculty of Medicine, Justus-Liebig-University, Friedrichstr. 24, 35392 Giessen, Germany; E-Mails: nils.bayer@yahoo.de (N.B.B.); uwe.schubert@biochemie.med.uni-giessen.de (U.S.); zehra.sentuerk@kmub.th-mittelhessen.de (Z.S.); sandra.frank@biochemie.med.uni-giessen.de (S.F.); hildegard.geyer@biochemie.med.uni-giessen.de (H.G.); rudolf.geyer@biochemie.med.uni-giessen.de (R.G.); klaus.t.preissner@biochemie.med.uni-giessen.de (K.T.P.); 2 Institute of Nutritional Science, Justus-Liebig-University, Wilhelmstr. 20, 35392 Giessen, Germany; E-Mail: silvia.rudloff@uni-giessen.de; 3 Institute of Organic Chemistry, Justus-Liebig University, Heinrich-Buff-Ring 58, 35392 Giessen, Germany; E-Mail: heike.hausmann@org.Chemie.uni-giessen.de

**Keywords:** glycoengineering, artificial sialic acids, mannosamine, angiogenesis

## Abstract

*N*-acetylneuraminic acid (Neu5Ac) represents the most common terminal carbohydrate residue in many mammalian glycoconjugates and is directly involved in a number of different physiological as well as pathological cellular processes. Endogenous sialic acids derive from the biosynthetic precursor molecule *N*-acetyl-D-mannosamine (ManNAc). Interestingly, *N*-acyl-analogues of D-mannosamine (ManN) can also be incorporated and converted into corresponding artificial sialic acids by eukaryotic cells. Within this study, we optimized a protocol for the chemical synthesis of various peracetylated ManN derivatives resulting in yields of approximately 100%. Correct molecular structures of the obtained products ManNAc, *N*-propanoyl-ManN (ManNProp) and *N*-butyl-ManN (ManNBut) were verified by GC-, ESI-MS- and NMR-analyses. By applying these substances to human umbilical vein endothelial cells (HUVECs), we could show that each derivative was metabolized to the corresponding *N*-acylneuraminic acid variant and subsequently incorporated into nascent glycoproteins. To investigate whether natural and/or artificial sialic acid precursors are able to modulate the angiogenic capacity of HUVECs, a spheroid assay was performed. By this means, an increase in total capillary length has been observed when cells incorporated *N*-butylneuraminic acid (Neu5But) into their glycoconjugates. In contrast, the natural precursor ManNAc inhibited the growth of capillaries. Thus, sialic acid precursors may represent useful agents to modulate blood vessel formation.

## 1. Introduction

Sialic acids are commonly found as terminal residues of carbohydrate moieties of both glycoproteins as well as glycolipids [1,2,3]. During the last decades, many studies have elucidated these acidic monosaccharides to be crucially involved in different biological processes [4]. In this context, for example, sialylLewisX-dependent homing of leukocytes via selectins represents one of the best characterized mechanisms [5].

**Figure 1:**
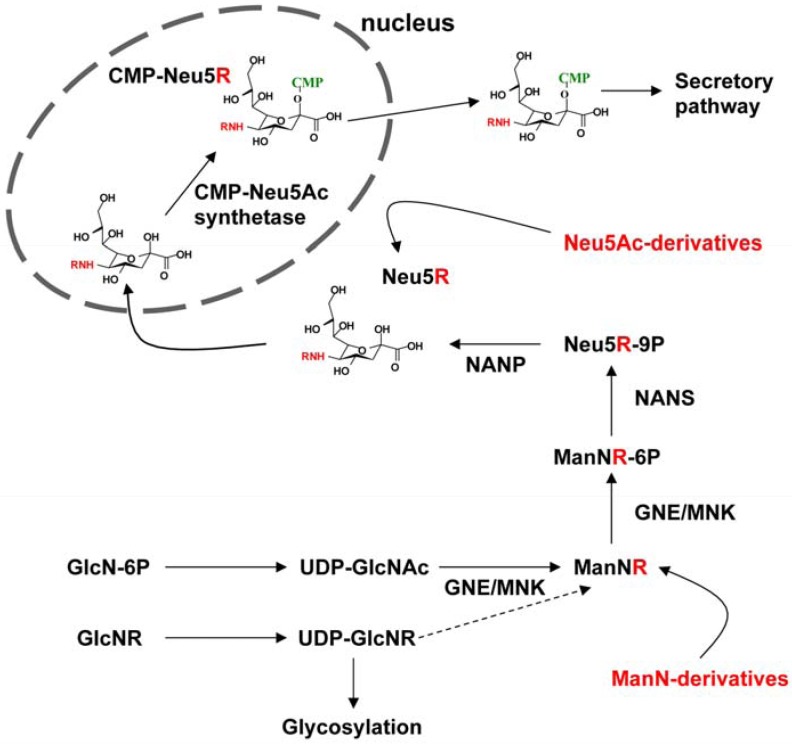
Biosynthetic pathway of sialic acids and feasible implementations of artificial precursors. R, acyl chains; GNE/MNK, uridinediphospho-*N*-acetyl-glucosamine-2-epimerase/N-acetylmannosamine kinase; NANS, *N*-acetylneuraminic acid synthase; NANP, *N*-acylneuraminate-9-phosphatase.

Endogenous biosynthesis of *N*-acetylneuraminic acid (Neu5Ac) is based on the phosphorylation of the precursor molecule *N*-acetyl-D-mannosamine (ManNAc) and its subsequent conjugation with pyruvate (Figure 1) [1,2,3]. Subsequent activation of Neu5Ac takes place in the nucleus resulting in the formation of CMP-Neu5Ac. After its active transport into the Golgi network, biosynthesis of sialylated glycoconjugates is completed by transfer of Neu5Ac to both nascent glycoproteins and glycolipids. Intriguingly, enzymes involved in the cellular sialic acid metabolism are also known to metabolize artificial sialic acid intermediates that possess elongated acyl chains at their amino groups. Thus, metabolic glycoengineering represents a powerful strategy to modulate interactions which depend upon the presence of sialic acids [6].

So far, multiple approaches have been exploited to insert *N*-acylneuraminic acids (Neu5R) into various cell lines (Figure 1) [7]. Due to the substrate specificity of the key enzyme uridinediphospho-*N*-acetylglucosamine-2-epimerase/*N*-acetylmannosamine kinase (GNE/MNK), that displays a striking specificity for physiological UDP-GlcNAc, chemically modified glucosamine analogues ended up to be poor candidates for sialo-engineering [6]. An alternative strategy is represented by the use of Neu5Ac or CMP-Neu5Ac analogues, since these components do not have to undergo substantial structural changes. However, their synthesis is not standardized yet, and, moreover, sialic acids are very expensive. Further studies indicated D-mannosamine (ManN) to be a more suitable target for chemical modification, since it is the common precursor of all sialic acids, it is very stable and can be easily modified [8]. Furthermore, overall cellular sialylation is mainly regulated by allosteric feedback inhibition of GNE [6]. Since addition of *N*-acyl-ManN evades this checkpoint, levels of artificial sialic acids increase proportional to the amount of ManN derivatives applied [7].

In terms of their biological functions, sialic acids on endothelial cells were also reported to influence the initiation of lumen formation in developing blood vessels [9]. To investigate the impact of artificial sialic acids on capillary sprouting, human umbilical vein endothelial cells (HUVECs) were treated with ManNAc or artificial sialic acid precursors and respective capillary sprouting was analyzed after stimulation with basic fibroblast growth factor (bFGF).

## 2. Results and Discussion

### 2.1. Synthesis of Artificial Sialic Acid Precursors

**Figure 2:**
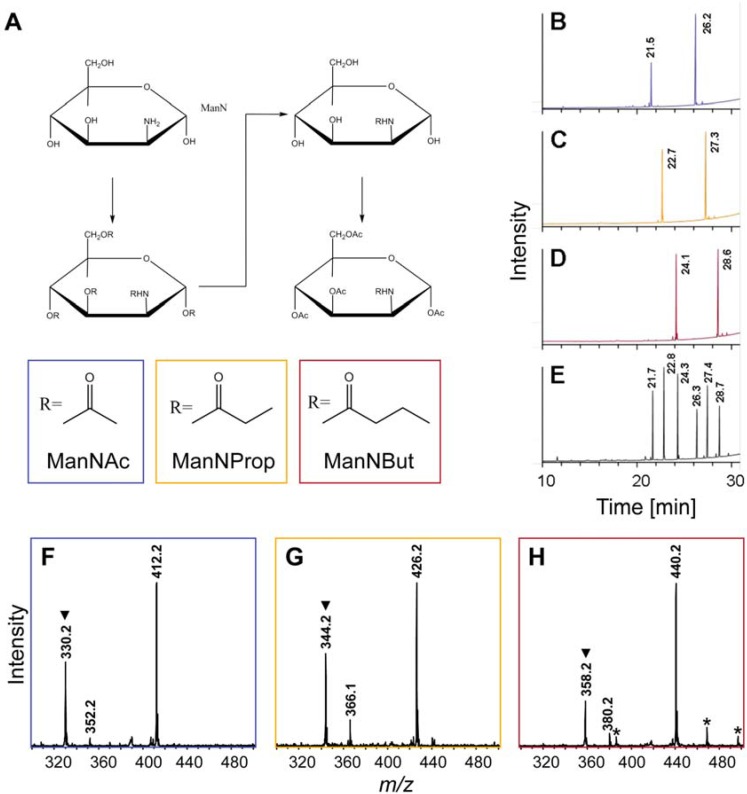
Chemical synthesis of peracetylated *N*-acyl-ManN derivatives. (**A**) Reaction sequence including peracylation, cleavage of *O*-acyl-residues and peracetylation. GC analyses were performed to confirm the synthesized structures to be peracetylated (**B**) ManNAc, (**C**) ManNProp, and (**D**) ManNBut by comparing respective retention times to those of (**E**) standard substances, which were confirmed by NMR analysis. Further structural verification was performed by ESI-MS analyses of peracetylated (**F**) ManNAc, (**G**) ManNProp, and (**H**) ManNBut registered as sodium adducts ([M+Na]^+^) as well as sodium adducts after the loss of acetic acid. Hydrogen adducts ([M+H]^+^) formed after the loss of acetic acid are marked by trianglels (▼) and unidentified signals are labeled by asterisks (*). Retention times as well as monoisotopic masses are assigned with the first decimal place.

ManNAc, *N*-propionyl-ManN (ManNProp) and *N*-butyl-ManN (ManNBut) were generated on the basis of published protocols [8]. However, we divided the synthesis into three independent reaction steps to increase the overall yields (Figure 2A), since in our hands only 50% of ManN was converted into the respective artificial sialic acid precursors, when the published protocol was employed. At first, ManN was incubated with respective anhydrides of acetic acid, propionic acid and butyric acid resulting in the modification of both the hydroxyl groups and the amino group. Subsequently, mild alkaline conditions removed the acyl chains from the hydroxyl groups leaving the modified amino residue untouched. Finally, addition of acetic acid anhydride resulted in the generation of per-*O*-acetylated ManNR derivatives that are able to penetrate cellular membranes due to their highly hydrophobic characteristics. Based on the presence of nonspecific cytoplasmatic esterases, *O*-acetyl-groups are cleaved resulting in the formation of respective *N*-acyl-ManN derivatives.

The accurate synthesis of artificial sialic acids was verified by gas chromatography (GC, Figure 2B–E), electrospray ionization-mass spectrometry (ESI-MS, Figure 2F–H), and NMR analyses (cf. Experimental Section). In comparison to retention times of standard substances, GC profiles of respective *N*-acyl-ManN derivatives revealed the presence of the α-anomer (first peak) as well as the β-anomer (second peak) of per-*O*-acetylated ManNAc, ManNProp and ManNBut (Figure 2B–D), thus demonstrating the successful synthesis of all derivatives to work under the conditions described. Corresponding ESI-MS analyses resulted in the predominant detection of sodium adducts ([M+Na]^+^) of particular derivatives at *m/z* 412.2, 426.2 and 440.2 (Figure 2F–H). Further fragmentation of selected adducts in ESI-MS^2^ experiments verified the annotations described above (data not shown).

By using this step-by-step protocol for the synthesis of artificial sialic acids, no additional purification steps were needed. The protocol has been proven to work well for amounts of up to 250 µg per preparation and the calculated yield was approximately 100%. The given values for temperature and pH as well as anhydrous conditions, however, have to be followed closely during the entire synthesis procedure. Otherwise, an epimerization of ManN into glucosamine takes place preventing successful synthesis of *N*-acyl-ManN derivatives (data not shown).

### 2.2. Integration of Artificial Sialic Acids into Glycoconjugates in HUVECs

**Figure 3:**
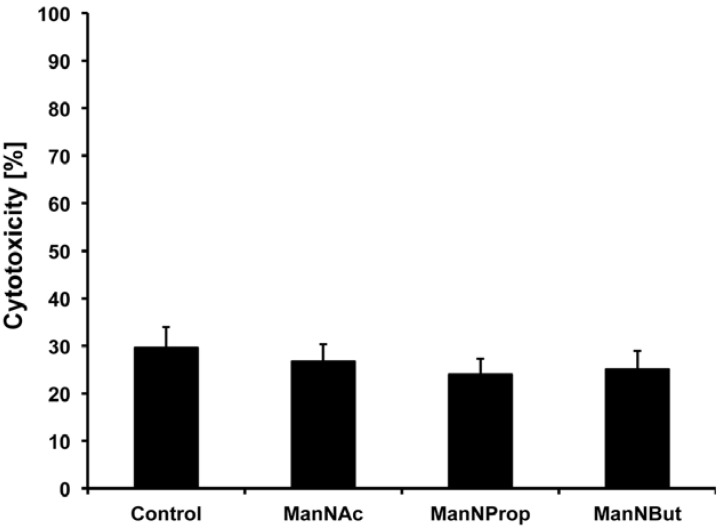
Biocompatibility of peracetylated ManNProp, ManNBut, and ManNAc. HUVECs were incubated with 50 µM of per-*O*-acetylated ManNProp, ManNBut, and ManNAc for 48 h and supernatants were checked for LDH activity. 100% was set for Triton-X 100 (1% v/v) treated HUVECs. All values ± S.D. in this figure are means of 3 independent experiments.

The synthesized substances were applied to the cell culture medium of HUVECs with a final concentration of 50 µM per ManNR derivative. At first, the biocompatibility of all generated components was determined. For this purpose, the release of lactate dehydrogenase (LDH) was compared with untreated cells. In agreement with other studies, concentrations of 50 µM had no significant effect on the cell behavior (Figure 3) allowing a treatment of HUVECs with all ManNR derivatives [10,11].

**Figure 4:**
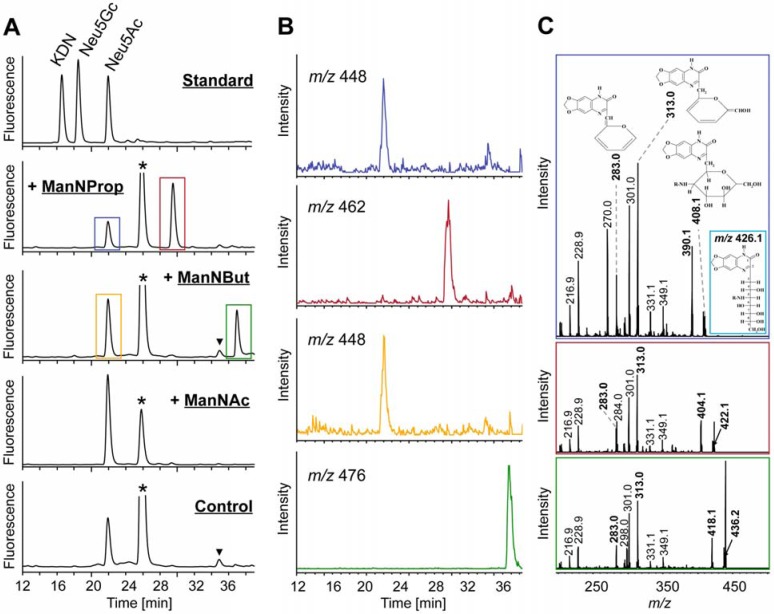
Analysis of sialic acids present in whole cell lysate from HUVECs treated with peracetylated ManNProp, ManNBut, and ManNAc. (**A**) DMB-labeled sialic acid residues were separated by RP-HPLC. For control, HUVECs were not incubated with any sialic acid precursors. (**B**) EIC of sodium adducts ([M+Na]^+^) of Neu5Ac (*m/z* 448) and Neu5Prop (*m/z* 462) as well as Neu5Ac (*m/z* 448) and Neu5But (*m/z* 476) present in HUVECs incubated with peracetylated ManNProp and ManNBut, respectively. Peaks related to DMB reagent are labeled with an asterisk (*) and those related to further unidentified impurities are marked with a triangle (▼). (**C**) ESI-MS/MS analyses of Neu5Ac (upper profile), Neu5Prop (central profile) and Neu5But (lower profile) are registered as proton adducts ([M+H]^+^). For fragmentation analyses, respective parent ions generated after loss of water were selected. Annotation of fragment ions was performed according to the fragmentation pathway of DMB-labeled sialic acids proposed by Manzi and co-workers [12] with monoisotopic masses of respective fragment ions printed in bold. Corresponding structures of generated fragments are given exemplarily in the upper profile. For the structure of Neu5Ac (*m/z* 426.1) prior to loss of water see inset.

Using a LC-ESI-MS(/MS) approach of total cell lysate we wanted to analyze whether each derivative was converted into the corresponding Neu5R analogue (Figure 4). For this purpose, cells were homogenized, sialic acids present were fluorescently labeled with 1,2-diamino-4,5-methylene-dioxybenzene (DMB) and generated reaction products were separated by RP-HPLC (Figure 4A). Comparison of retention times of registered signals with those of standard substances revealed Neu5Ac to be also present in HUVECs incubated with ManNProp and ManNBut (marked with a blue and yellow rectangle, respectively). In addition, peaks at 29.5 min (red rectangle) and 37.0 min (green rectangle) indicated the presence of Neu5Prop and Neu5But, respectively. Cells incubated exclusively with ManNAc provided a single signal for Neu5Ac. Extracted ion chromatograms (EIC) of *m/z* values corresponding to sodium adducts ([M+Na]^+^) of respective artificial sialic acids confirmed the presence of Neu5Ac (*m/z* 448) and Neu5Prop (*m/z* 462) as well as Neu5But (*m/z* 476) (Figure 4B). ESI-MS/MS analyses of selected precursor ions registered as hydrogen adducts ([M+H]^+^) provided the characteristic fragmentation pattern of DMB-labeled sialic acids as proposed elsewhere [11,12] (Figure 4C, respective signals printed in bold). Sialic acid precursor ions formed after loss of water were selected for these fragmentation analyses (Neu5Ac-H_2_0, *m/z* 408.1; Neu5Prop-H_2_0, *m/z* 422.1 and Neu5But-H_2_0, *m/z* 436.2).

Exemplarily, the structure of Neu5Ac prior to loss of water is given in the inset of the upper profile. Next to the detection of fragment ions of identical *m/z* values (*m/z* 216.9, 228.9, 283.0, 301.0, 313.0, 331.1 and 349.1), whose generation was based on fragmentation pathways within which the *N*-acyl side chain is lost, signals at *m/z* 270.0 in case of Neu5Ac and corresponding signals at *m/z* 284.0 and 298.0 in case of Neu5Prop and Neu5But indicated cross-ring fragmentations within the DMB moiety, thus leaving the *N*-acyl-side chain of respective artificial sialic acids intact. In a similar way, fragment ions at *m/z* 390.1, 404.1 and 418.1 reflected the loss of water from respective parent ions.

**Figure 5:**
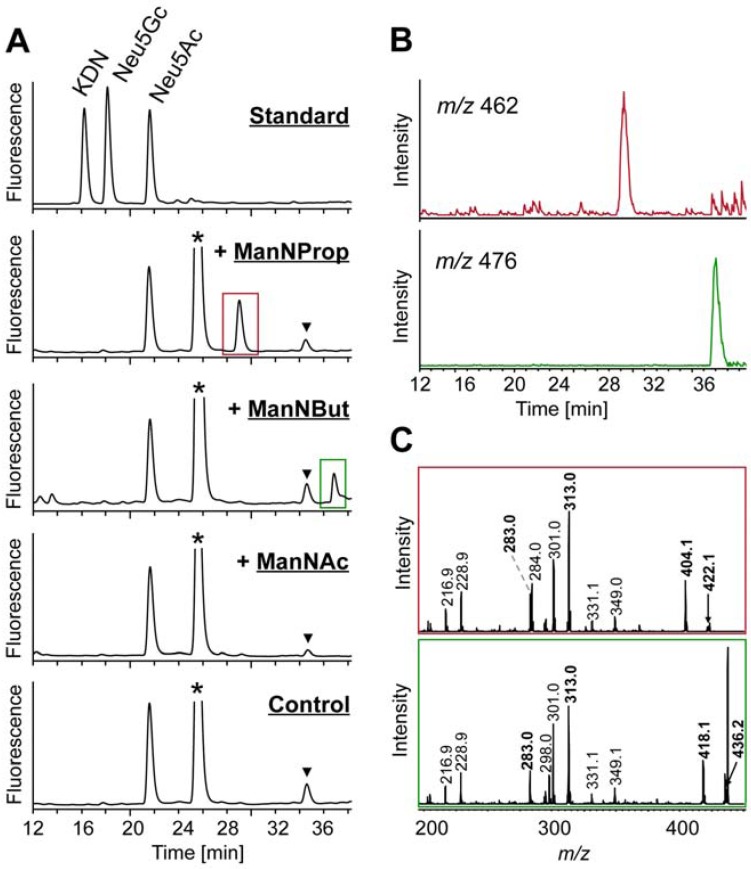
Analysis of sialic acid residues of glycoproteins isolated from HUVECs after incubation with per-*O*-acetylated ManNProp, ManNBut, and ManNAc. (**A**) Sialic acid residues obtained after DMB-labeling were separated by RP-HPLC. For control, HUVECs were not incubated with any sialic acid precursors. Peaks related to DMB reagent are labeled with an asterisk (*) and those related to unknown impurities are marked with a triangle (▼). (**B**) EIC of sodium adducts ([M+Na]^+^) of Neu5Prop (*m/z* 462) and Neu5But (*m/z* 476) present in glycoproteins from HUVECs incubated with ManNProp and ManNBut, respectively. (**C**) ESI-MS/MS analyses of Neu5Prop (upper profile) and Neu5But (lower profile) registered as proton adducts ([M+H]^+^). Annotation of fragment ions was carried out as defined in Figure 4.

These results revealed that chemically synthesized peracetylated ManNProp and ManNBut were incorporated by HUVECs and subsequently transformed into Neu5Prop and Neu5But, respectively. To determine whether these artificial sialic acids are also incorporated into nascent glycoproteins, HUVECs were incubated with ManNAc, ManNProp and ManNBut followed by isolation of glycoproteins and the analysis of the attached sialic acid residues (Figure 5). RP-HPLC analyses of DMB-labeled sialic acids present in cellular glycoproteins provided equal results compared to respective analyses of total cell lysates (Figure 5A; cf. Figure 4A). Peaks representing Neu5Prop and Neu5But were detectable at 29.0 min and 36.8 min, respectively. ESI-MS(/MS) identified these Neu5R analogues by registration of their sodium adducts ([M+Na]^+^) in EIC of *m/z* 462 and 476 (Figure 5B) as well as by displaying the characteristic fragmentation pattern of respective precursor molecules (Figure 5C). These findings demonstrated that incorporation of artificial sialic acid precursors into glycoproteins takes place resulting in an alteration of cell sialylation *in vitro*.

### 2.3. Artificial Sialic Acids Influence Capillary Sprouting of HUVECs

**Figure 6:**
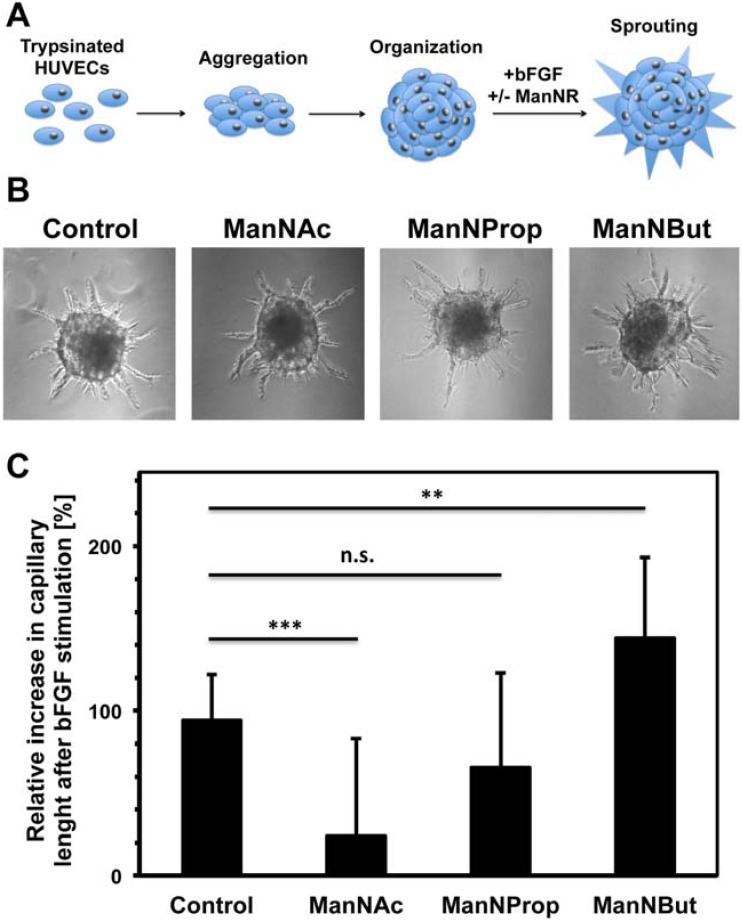
Influence of per-*O*-acetylated ManNR derivatives on capillary sprouting of endothelial cell spheroids induced by bFGF. (**A**) Scheme of the applied three dimensional angiogenesis assay. HUVECs spheroids were treated with bFGF to induce capillary sprouting in absence (control) or presence of indicated sialic acid precursor molecules (R, acyl chains) [13,14]. (B) Representative spheroids after bFGF stimulation. (**C**) The lengths of arising capillaries were measured and obtained values were summed up using NIS Elements AR 3.0 Software (Nikon, Düsseldorf, Germany). Results are shown as relative increase in total capillary length after stimulation with bFGF. To this end, total capillary lengths of spheroids were measured after bFGF stimulation and compared with the unstimulated control. All values ± S.D. in this figure are means of 3 independent experiments using at least 12 spheroids per set. The statistical evaluation was performed by Student´s *t* test (unequal variances, two tailed). Significance levels are indicated by n.s. (not significant), *p* > 5%; *, *p* < 5%; **, *p* < 1%; ***, *p* < 0.1%.

By use of a spheroid assay we wanted to elucidate whether the observed alteration in cell sialylation also affects physiological functions of endothelial cells such as vessel formation. To this end, cells were incubated with per-*O*-acetylated ManNR derivatives, stimulated with bFGF and arising capillary lengths were measured and compared with unstimulated spheroids (Figure 6). Surprisingly, the results demonstrated that sprouting of spheroids–treated with the natural sialic acid precursor ManNAc–was significantly inhibited. ManNAc caused a reduction of total capillary length by 75% of the values obtained for spheroids, which were not co-stimulated with sialic acid precursors. After ManNProp treatment, however, the sprouting capability of HUVEC spheroids was similar to the control spheroids. Intriguingly, ManNBut treatment significantly enhanced capillary sprouting of HUVECs after bFGF stimulation. The total capillary length was 50% higher in comparison to spheroids, which were not incubated with any ManNR derivate.

The relative increase in total capillary length in endothelial cell spheroids incubated with ManNBut might be induced by its structural differences compared to ManNAc. Therefore, one may suggest that the additional ethylene group in its elongated acyl-chain impedes the interaction of Neu5But with accessory enzymes or carbohydrate-binding receptors, which are involved in the formation of blood vessels. For example, glycosylation of integrins and fibroblast growth factor receptors is essential for their biological functions [15,16,17]. Consequently, not only the negative charge of sialic acids might be necessary for the regulation of blood vessel formation as already mentioned before [9], but also a specific recognition of Neu5Ac could be involved.

In addition to glycoproteins, however, formation and relative proportions of different gangliosides like GD3 and GM3 are important for angiogenic processes and might be disturbed by administration of ManNAc and/or artificial sialic acid precursors [18,19]. In the case of GD3, for example, an α2,8-sialyltransferase activity is necessary for the generation of this ganglioside. Other groups could show that some α2,8-sialyltransferases can be stimulated by ManNAc or inhibited by ManNProp and ManNBut [20,21].

Apart from the sialome of the HUVEC surface, also intracellular signaling might be influenced by the application of ManNR derivates. The intracellular CMP-Neu5Ac level is discussed to affect cell proliferation, gene expression as well as phosphorylation of intracellular signaling molecules like the extracellular signal-regulated kinase ERK [22]. The impact of artificial CMP-Neu5R derivatives, however, has not been addressed in this context so far. Furthermore, ManNR can be partially converted to GlcNR [23]. Thus, intracellular O-GlcNAc-ylation might be also changed leading to an inhibition of phosphorylation [24]. Taken together, we could show that ManNR derivatives influence the angiogenic capacity of HUVECs. The precise role of the applied substances during sprouting processes, however, needs to be further investigated.

## 3. Experimental

### 3.1. Chemicals and Reagents

The monosaccharide standards ManN, Neu5Ac, Neu5Gc and 2- keto-3-deoxy-d-glycero-d-galacto-nononic acid (KDN) were purchased from Sigma-Aldrich (Taufkirchen, Germany). All reagents used were of analytical grade.

### 3.2. Synthesis of Ac4-ManN Derivatives

ManN hydrochloride (250 µg) was peracylated by incubation with pyridine (100 µL) and respective carbonic acid anhydride (acetic acid, propionic acid and butyric acid for the synthesis of ManNAc, ManNProp and ManNBut, respectively, 300 µL) at 37 °C overnight. To specifically remove *O*-acyl-chains while maintaining *N*-acylation, samples were dried down under a stream of nitrogen and dissolved in 0.01 M methanolic NaOH (500 µL) at 0 °C for 2 h. Subsequently, samples were frozen in liquid nitrogen and lyophilized overnight. Peracetylation of hydroxyl-groups was achieved by incubation with pyridine (100 µL) and ethanoic anhydride (300 µL) overnight at room temperature.

### 3.3. Cell Culture

Isolation of HUVECs was carried out using the method described by Minick and co-workers [25] (approved by the local research ethics committee (No. 132/09). Cells were cultivated with endothelial cell growth medium (ECGM; Promocell, Heidelberg, Germany) containing 10% fetal calf serum (FCS; Thermo Fisher Scientific, Bonn, Germany) (v/v) in the absence or presence of sialic acid precursor molecules (50 mM). After three washing steps with PBS, cells were harvested using a cell scraper to protect surface glycoproteins from lysis by trypsine. Subsequently, cells were homogenized in 10 mM Tris/HCl-buffer (pH 8.0) containing 1% Triton-X 100 (v/v) using an ultrasonic dispersor. Cytotoxicity was determined with the LDH cytotoxicity assay (Roche Applied Science, Mannheim, Germany) according to the instructions of the manufacturer.

### 3.4. Sample Preparation for the Analysis of Sialic Acids

To distinguish between cytosolic sialic acid derivatives and those actually incorporated into glycoproteins one aliquot of the homogenized cells was dried down and subjected to sialic acid analysis. Remaining sample was mixed thoroughly with chloroform:methanol (1:2, v/v, 600 µL). After centrifugation, both the chloroform and the methanol phase were discarded. The obtained interphase, containing cellular proteins, was homogenized in ice cold 90% ethanol and dried for sialic acid analyses.

### 3.5. DMB-Labeling of Sialic Acids

For fluorescent labeling, samples were hydrolyzed in 2 M acetic acid (200 µL) for 90 min at 80 °C and dried down. Subsequently, samples were dissolved in DMB-reaction buffer [80 µL, 7 mM DMB (Dojindo, Kumamoto, Japan), 500 mM 2-mercaptoethanol, 9 mM sodium hydrosulfite, 20 mM trifluoroacetic acid (TFA)] and incubated for 2 h at 55 °C [26,27]. Reaction was stopped by adding 0.2 M NaOH (20 µL).

### 3.6. HPLC-Separation

DMB-labeled samples were analyzed on a Superspher 100 C-18 column (250 mm × 40 mm, Merck-Hitachi, Darmstadt, Germany) at 40 °C using a Knauer HPLC system. Mobile phases M1 [methanol/acetonitrile(AcN)/water/TFA = 4:4:92:0.1 (v/v/v)] and M2 [methanol/AcN/water/TFA = 45:45:10:0.1, v/v/v) were used for their separation. A linear gradient was applied from 0% to 100% M2 in 35 min at a flow rate of 0.25 mL/min. A fluorescence detector (Merck-Hitachi, Darmstadt, Germany) was set at 372 nm for excitation and 456 nm for emission.

### 3.7. GC (-MS) Analyses

Samples were separated on a 60 m RTx 5MS column (Restek GmbH, Bad Homburg Germany) using a Varian Star 3400 gas chromatograph (Agilent, Santa Clara, CA, USA). To this end, samples were injected manually at an injector temperature of 270 °C. Starting from an initial column temperature of 130 °C for 1 min, a temperature gradient of 5 °C/min was applied up to a maximum temperature of 290 °C, which was maintained for 10 min before cooling down. Detection was carried out by a flame ionization detector at 300 °C. GC-MS analyses were performed on a Polaris Q instrument (Thermo Scientific, Dreieich, Germany). To this end, samples were separated on a 60 m VF 5MS column (Agilent) using the following conditions: T_0 min_ = 50 °C, T_1.88 min_ = 50 °C, T_4.63 min_ = 160 °C, T _28.63 min_ = 280 °C, and T_ 30 min_ = 280 °C. For GC and GC-MS analyses helium 5.6 was used as carrier gas. GC and GC-MS data were processed and analyzed with EuroChrom Software (Knauer) and Xcalibur Software (Thermo Scientific), respectively.

### 3.8. nanoLC-ESI-MS(/MS)-Analysis

Ten µL of sample was separated on a RP-column (PepMap, 3 µm spheres 75 µm × 100 mm, LC Packings) using an Ultimate nanoLC system. Mobile phases M1 [AcN/water/formic acid = 8:92:0.1 (v/v/v)] and M2 [AcN/water/formic acid = 90:10:0.1 (v/v/v)] were used for separation with a linear gradient from 0% to 20% M2 in 30 min at a flow rate of 0.3 µL/min. The Ultimate nanoLC system was directly coupled with an Esquire 3000 ESI-ion trap-MS (Bruker Daltonics, Bremen, Germany) operated with spray voltage of 1.4 kV, capillary temperature of 250 °C, end plate offset of -500V, and capillary exit of 140V. Recorded MS data were processed and analyzed using DataAnalysis Software (Bruker Daltonics).

### 3.9. NMR-Analysis

The molecular structures of the obtained products ManNAc, *N*-propanoyl-ManN (ManNProp) and *N*-butyl-ManN (ManNBut) were verified by ^1^H-NMR spectroscopy. To obtain the complete ^1^H chemical shift assignments the structure elucidation was based on the application of homonuclear ^1^H,^1^H correlation spectroscopy (COSY and TOCSY) and heteronuclear ^1^H,^13^C-correlation spectroscopy (edited-HSQC and HMBC). All one-dimensional and two-dimensional NMR experiments were performed on a Bruker Avance III 600 spectrometer equipped with a 5 mm BBO Z-gradient probe.The data were collected and processed by TOPSPIN software (Bruker) running on a PC with Microsoft Windows^XP^. The NMR experiments were performed using Bruker standard pulse sequences and parameters. Chemical shifts are reported in ppm (δ scale) using the solvent signal as standard and coupling constants (J) are reported in Hz. Multiplicities of NMR signals are designated as s (singlet), d (doublet), t (triplet), q (quartet), br (broad), m (multiplet, for unresolved lines), *etc.* ManNAc, *N*-propanoyl-ManN (ManNProp) and *N*-butyl-ManN (ManNBut) gave acceptable ^1^H-NMR spectra that matched the data reported in the cited references [15,16,17]. The one-bond ^13^C, ^1^H coupling constant ^1^*J*(^13^C,^1^H) for the anomeric proton was used as a supplement to the chemical shift data for the assignment of the anomeric structure of ManNAc and ManNProp [18].

*ManNAc*.^ 1^H-NMR (acetone-d_6_): δ (mixtures of anomers) (α-isomer) δ 7.59 (d, 1H, *J*_NH,H-2_ 9.2 Hz, NH), 5.97 (d, 1H, *J*_1,2_ 1.8 Hz, ^1^*J*_H-1,C-1_ 177.6 Hz, H-1), 5.27 (m, 1H, H-3), 5.23 (m, 1H, H-4), 4.58 (m, 1H, H-2), 4.01–4.15 (m, 2H, H-6,6′), 4.10 (m, 1H, H-5); (β-isomer) 7.40 (d, 1H, *J*_NH,H-2 _9.5Hz, NH), 5.97 (d, 1H, J_1,2_ 1.9 Hz, ^1^*J*_H-1,C-1_ 166.7 Hz, H-1), 5.16 (m, 1H, H-3), 5.14 (m, 1H, H-4), 4.77 (m, 1H, H-2), 4.06–4.17 (m, 2H, H-6,6′), 3.97 (m, 1H, H-5), 2.20–1.90 (s, -CH_3_, Ac).

*N-Propanoyl-ManN (ManNProp)*. ^1^H-NMR (acetone-d_6_): δ (mixtures of anomers) (α-isomer) δ7.48 (d, 1H, *J*_NH,H-2_ 8.8 Hz, NH), 5.96 (d, 1H, *J*_1,2_ 1.8 Hz, ^1^*J*_H-1,C-1_ 176.6 Hz, H-1), 5.28 (m, 1H, H-3), 5.26 (m, 1H, H-4), 4.60 (m, 1H, H-2), 4.02–4.17 (m, 2H, H-6,6′), 4.10 (m, 1H, H-5), 2.25 (q, -CH_2_-, Pr), 2.17, 2.04, 1.98, 1.93 (s, -CH_3_, Ac), 1.07 (t, -CH_3_, Pr); (β-isomer) 7.29 (d, 1H, *J*_NH,H-2 _9.8 Hz, NH), 5.98 (d, 1H, J_1,2_ 2.0 Hz, ^1^*J*_H-1,C-1_ 167.8 Hz, H-1), 5.17 (m, 1H, H-3), 5.16 (m, 1H, H-4), 4.79 (m, 1H, H-2), 4.07–4.19 (m, 2H, H-6,6′), 3.92 (m, 1H, H-5), 2.25 (q, -CH_2_-, Pr), 2.17, 2.04, 1.98, 1.93 (s, -CH_3_, Ac), 1.07 (t, -CH_3_, Pr).

*N-Butyl-ManN (ManNBut)*. ^1^H-NMR (acetone-d_6_): δ 7.30 (d, 1H, *J*_NH,H-2 _9.4 Hz, NH), 5.98 (d, 1H, *J*_1,2_ 1.9 Hz, H-1), 5.18 (m, 1H, H-3), 5.17 (m, 1H, H-4), 4.79 (m, 1H, H-2), 4.06–4.18 (m, 2H, H-6,6′), 3.98 (m, 1H, H-5), 2.21 (t, 2H, -CH_2_-, Bu), 2.04, 2.03, 2.00, 1.92 (s, 3H, Ac), 1.63 (m, 2H, -CH_2_-, Bu), 0.93 (t, 3H, -CH_3_, Bu).

### 3.10. Spheroid Assay

Endothelial cell spheroids were generated as described by Korff and Augustin [16]. To this end, HUVECs were suspended in ECGM containing 20% methylcellulose (w/v) (Sigma-Aldrich, München, Germany) and 3,000 cells *per* well were cultivated under non-adhesive conditions in round bottom 96-well plates (Greiner Bio One, Frickenhausen, Germany) for 18–24 h at 37 °C. Spheroid assay was performed according to the micro-bead angiogenesis protocol as described previously [16,17].

Fibrin matrix was prepared as described by Orci and co-workers [17]. Bovine fibrinogen (Calbiochem/Merck Millipore, Darmstadt, Germany) was dissolved in PBS, pH 7.4. This stock solution was adjusted to a final concentration of 1.8 mg/mL in Dulbeccos PBS, pH 7.4 (Invitrogen, Karlsruhe, Germany), and 200 U/mL of protease inhibitor Trasylol (Bayer, Leverkusen, Germany) were added. Spheroids were harvested, washed and a sufficient volume was transferred to the fibrinogen solution in 24-well plates (Corning, Wiesbaden, Germany). Fibrinogen was polymerized by the addition of 0.65 U/mL thrombin (Sigma-Aldrich). Gels were equilibrated for 30 min at 37 °C with endothelial basal medium (Promocell) containing 0.5% (v/v) FCS. On replacement with fresh medium, test substances together with bFGF were added and the plates were incubated for 48–72 h at 37 °C. Subsequently, cells were fixed with 4% paraformaldehyde (w/v) and stored at 4 °C.

## 4. Conclusions

The outermost position of sialic acids in glycoproteins and glycolipids makes them accessible to numerous cellular communication elements involved in the formation of contacts with components of the same or neighboring cell, with extracellular matrix or invading pathogens [4]. So far, many studies have provided evidence that the application of artificial sialic acids represents a promising tool to modulate such sialic acid-dependent processes. For example, elongated *N*-acyl side chains of sialic acids have been shown to provide protection against an infection with influence virus A *in vitro* [28]. Recently, it has also been demonstrated that the presence of artificial sialic acids on glycoproteins may affect their resistance to sialidases [29]. Thus, biological half-life of therapeutic glycoproteins might be efficiently increased also *in vivo*.

In this study, we describe an optimized protocol for the synthesis of artificial sialic acid precursors and, furthermore, demonstrate that endothelial cells metabolize these glycoengineered monosaccharides and incorporate the resulting modified sialic acids into their cellular glycoproteins. Intriguingly, natural sialic acid precursors inhibited the angiogenic capacity of HUVECs. Hence, administration of natural sialic acid precursors might be a useful tool to inhibit angiogenesis in cancer patients. In contrast, Neu5But precursors led to an extended bFGF-mediated capillary sprouting of HUVECs. Thus, ManNBut could represent a suitable agent to stimulate blood vessel formation during, e.g., wound healing processes. However, further *in vitro* tests including a migration assay, vascular endothelial growth factor (VEGF) induced sprouting and tube formation assays as well as *in vivo* models are required to evaluate the biomedical potential of ManNR derivates during angiogenic processes and to study the exact mechanisms leading to the observed modulation of angiogenesis.

Taken together, sialic acid glycoengineering offers numerous possibilities to influence physiological processes, but the resulting biological activities have to be carefully assessed to exclude undesired side effects.

## References

[1] Angata T., Varki A. (2002). Chemical diversity in the sialic acids and related alpha-keto acids: An evolutionary perspective. Chem. Rev..

[2] Schauer R. (2000). Achievements and challenges of sialic acid research. Glycoconj. J..

[3] Schauer R. (2004). Sialic acids: Fascinating sugars in higher animals and man. Zoology (Jena).

[4] Schauer R. (2009). Sialic acids as regulators of molecular and cellular interactions. Curr. Opin. Struct. Biol..

[5] McEver R.P., Moore K.L., Cummings R.D. (1995). Leukocyte trafficking mediated by selectin-carbohydrate interactions. J. Biol. Chem..

[6] Du J., Meledeo M.A., Wang Z., Khanna H.S., Paruchuri V.D., Yarema K.J. (2009). Metabolic glycoengineering: Sialic acid and beyond. Glycobiology.

[7] Keppler O.T., Horstkorte R., Pawlita M., Schmidt C., Reutter W. (2001). Biochemical engineering of the N-acyl side chain of sialic acid: Biological implications. Glycobiology.

[8] Keppler O.T., Stehling P., Herrmann M., Kayser H., Grunow D., Reutter W., Pawlita M. (1995). Biosynthetic modulation of sialic acid-dependent virus-receptor interactions of two primate polyoma viruses. J. Biol. Chem..

[9] Strilic B., Eglinger J., Krieg M., Zeeb M., Axnick J., Babal P., Muller D.J., Lammert E. (2010). Electrostatic cell-surface repulsion initiates lumen formation in developing blood vessels. Curr. Biol..

[10] Aich U., Campbell C.T., Elmouelhi N., Weier C.A., Sampathkumar S.G., Choi S.S., Yarema K.J. (2008). Regioisomeric SCFA attachment to hexosamines separates metabolic flux from cytotoxicity and MUC1 suppression. ACS Chem. Biol..

[11] Galuska S.P., Geyer H., Weinhold B., Kontou M., Rohrich R.C., Bernard U., Gerardy-Schahn R., Reutter W., Munster-Kuhnel A., Geyer R. (2010). Quantification of nucleotide-activated sialic acids by a combination of reduction and fluorescent labeling. Anal. Chem..

[12] Klein A., Diaz S., Ferreira I., Lamblin G., Roussel P., Manzi A.E. (1997). New sialic acids from biological sources identified by a comprehensive and sensitive approach: Liquid chromatography-electrospray ionization-mass spectrometry (LC-ESI-MS) of SIA quinoxalinones. Glycobiology.

[13] Korff T., Augustin H.G. (1998). Integration of endothelial cells in multicellular spheroids prevents apoptosis and induces differentiation. J. Cell Biol..

[14] Montesano R., Pepper M.S., Vassalli J.D., Orci L. (1987). Phorbol ester induces cultured endothelial cells to invade a fibrin matrix in the presence of fibrinolytic inhibitors. J. Cell Physiol..

[15] Sato Y., Isaji T., Tajiri M., Yoshida-Yamamoto S., Yoshinaka T., Somehara T., Fukuda T., Wada Y., Gu J. (2009). An *N*-glycosylation site on the beta-propeller domain of the integrin alpha5 subunit plays key roles in both its function and site-specific modification by beta1,4-*N*-acetylglucosaminyltransferase III. J. Biol. Chem..

[16] Isaji T., Sato Y., Fukuda T., Gu J. (2009). *N*-Glycosylation of the I-like domain of beta1 integrin is essential for beta1 integrin expression and biological function: Identification of the minimal *N*-glycosylation requirement for alpha5beta1. J. Biol. Chem..

[17] Duchesne L., Tissot B., Rudd T.R., Dell A., Fernig D.G. (2006). *N*-Glycosylation of fibroblast growth factor receptor 1 regulates ligand and heparan sulfate co-receptor binding. J. Biol. Chem..

[18] Manfredi M.G., Lim S., Claffey K.P., Seyfried T.N. (1999). Gangliosides influence angiogenesis in an experimental mouse brain tumor. Cancer Res..

[19] Ziche M., Morbidelli L., Alessandri G., Gullino P.M. (1992). Angiogenesis can be stimulated or repressed in vivo by a change in GM3:GD3 ganglioside ratio. Lab. Invest..

[20] Bork K., Gagiannis D., Orthmann A., Weidemann W., Kontou M., Reutter W., Horstkorte R. (2007). Experimental approaches to interfere with the polysialylation of the neural cell adhesion molecule *in vitro* and *in vivo*. J Neurochem.

[21] Horstkorte R., Muhlenhoff M., Reutter W., Nohring S., Zimmermann-Kordmann M., Gerardy-Schahn R. (2004). Selective inhibition of polysialyltransferase ST8SiaII by unnatural sialic acids. Exp. Cell Res..

[22] Weidemann W., Klukas C., Klein A., Simm A., Schreiber F., Horstkorte R. (2010). Lessons from GNE-deficient embryonic stem cells: Sialic acid biosynthesis is involved in proliferation and gene expression. Glycobiology.

[23] Luchansky S.J., Yarema K.J., Takahashi S., Bertozzi C.R. (2003). GlcNAc 2-epimerase can serve a catabolic role in sialic acid metabolism. J. Biol. Chem..

[24] Campbell C.T., Sampathkumar S.G., Yarema K.J. (2007). Metabolic oligosaccharide engineering: Perspectives, applications, and future directions. Mol. BioSyst..

[25] Jaffe E.A., Nachman R.L., Becker C.G., Minick C.R. (1973). Culture of human endothelial cells derived from umbilical veins. Identification by morphologic and immunologic criteria. J. Clin. Invest..

[26] Hara S., Takemori Y., Yamaguchi M., Nakamura M., Ohkura Y. (1987). Fluorometric high-performance liquid chromatography of *N*-acetyl- and *N*-glycolylneuraminic acids and its application to their microdetermination in human and animal sera, glycoproteins, and glycolipids. Anal. Biochem..

[27] Galuska S.P., Geyer H., Mink W., Kaese P., Kuhnhardt S., Schafer B., Muhlenhoff M., Freiberger F., Gerardy-Schahn R., Geyer R. (2012). Glycomic strategy for efficient linkage analysis of di-, oligo- and polysialic acids. J. Proteom..

[28] Keppler O.T., Herrmann M., von der Lieth C.W., Stehling P., Reutter W., Pawlita M. (1998). Elongation of the N-acyl side chain of sialic acids in MDCK II cells inhibits influenza A virus infection. Biochem. Biophys. Res. Commun..

[29] Werner A., Horstkorte R., Glanz D., Biskup K., Blanchard V., Berger M., Bork K. (2012). Glycoengineering the N-acyl side chain of sialic acid of human erythropoietin affects its resistance to sialidase. Biol. Chem..

